# The complete mitochondrial genome of the fig weevil, *Aclees cribratus* (Coleoptera: Curculionidae)

**DOI:** 10.1080/23802359.2020.1780978

**Published:** 2020-06-26

**Authors:** Bao-Xin Wang, Ying-Luo Xu, Zhi-Hang Zhuo, Xiu-Lan Xu, Ji Liu, Jing Qiu, Rui Fang, Yun-Ke Liu, Zhen Zeng, Qian-Gang Xiao

**Affiliations:** a Chengdu Academy of Agricultural and Forestry Sciences, Chengdu, Sichuan, China; b Key Laboratory of Ecological Forestry Engineering of Sichuan Province, College of Forestry, Sichuan Agricultural University, Chengdu, Sichuan, China; c College of Forestry, Hainan University, Haikou, Hainan, China

**Keywords:** Fig weevil, *Aclees cribratus*, mitochondrial genome

## Abstract

*Aclees cribratus* Gyllenhyl (Coleoptera: Curculionidae) is an important pest of fig. In this study, the complete mitogenome of *A. cribratus* was determined, which was 17,329 bp in length and contained 37 genes, including 13 protein-coding genes (PCGs), 2 rRNA, 22 tRNA genes, and 2 control regions. The phylogenetic analysis based on mitogenomes showed that *A. cribratus* is the sister group of Molytinae.

The fig weevil, *Aclees cribratus* Gyllenhyl (Coleoptera: Curculionidae) is an uncontrollable noxious insect and distributed widely in fig nursery (Ciampolini et al. 2005). The larvae of A. cribratus bore into the stem base of bole and the imagoes gnaws at shoots and fruits, causing serious damage to fig trees and productions. Adult specimens of *A. cribratus* were collected from Shuangliu District, Chengdu City, Sichuan Province, China (N30°19′14″, E104°01′18″) on 10 October 2019. The specimen was deposited in the insect specimen room of Chengdu Academy of Agricultural and Forestry Sciences (voucher no. D018008).

The circular mitochondrial genome of *A. cribratus* was 17,329 bp in length (GenBank accession number MT501538) and included sets of genes, including 13 protein-coding genes (PCGs), two ribosomal RNA genes (rRNAs), 22 transfer RNA genes (tRNAs), and 2 control regions located between *rrnS* and *trnI* gene, which were 224 bp and 58 bp. The gene order of *A. cribratus* were identical to other weevils mitogenomes (Zhang et al. 2019).

The nucleotide composition of the mitogenome was significantly biased (A, G, C, and T was 39.50%, 9.09%, 15.07%, and 36.34%, respectively), with A + T contents of 75.84%. The AT-skew and GC-skew of this genome were 0.042 and –0.247, respectively. Fourteen genes were transcribed on the J-strand, whereas the others were oriented on the N-strand. Gene overlaps were present at 14 gene junctions and involved a total of 57 bp, and the longest overlap (17 bp) existed between *trnF* and *nad5*. The structure and arrangement of tRNAs and rRNAs were similar to *Cyrotrachelus buqueti* (Yang et al. 2018).

The maximum likelihood tree (Zhang et al. 2020) was constructed based on the amino acid sequences of the 13 PCGs from the mitochondrial genomes of 19 Curculionidae species. The result supported that *A. cribratus* is the sister group of Molytinae (Figure 1).

**Figure 1.**
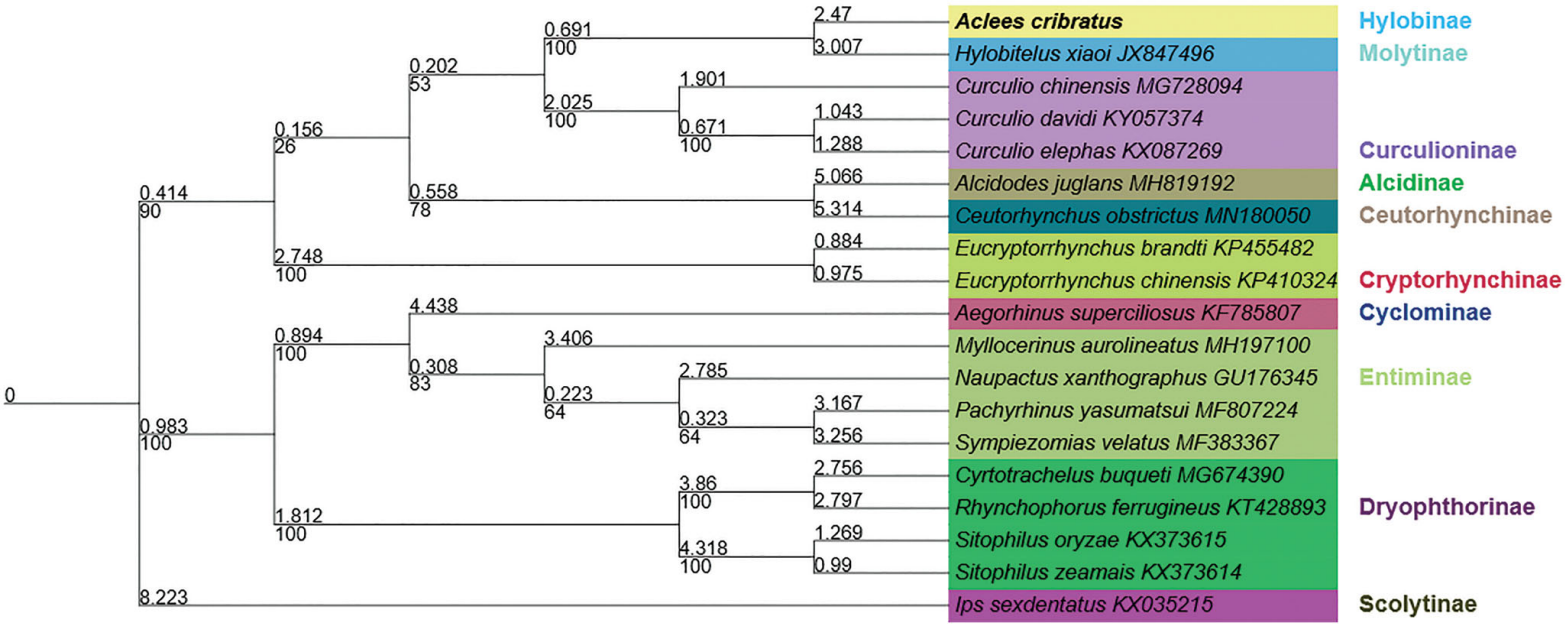
Maximum likelihood phylogenies were inferred using IQ-TREE under Edge-linked partition model for 5000 ultrafast bootstraps, approximate Bayes test, as well as the Shimodaira–Hasegawa-like approximate likelihood-ratio test. The colors represent different subfamilies. Branch lengths (above) and bootstrap values (below) were indicated around nodes. *Ips sexdentatus* was used as an outgroup. GeneBank accession numbers of each species were listed in the tree.

## Date availability statement

The data that support the findings of this study are openly available in the NCBI Genbank database at https://www.ncbi.nlm.nih.gov/genbank/, reference number MT501538。

## References

[CIT0001] Ciampolini M, Perrin H, Regalin R. 2005. *Aclees cribratus*, nuovo per I’Italia nocivo al fico allevato in vivaio. Informatore Agrario. 61(47):69–71.

[CIT0002] Yang WJ, Yang DX, Xu KK, Cao Y, Meng YL, Wu Y, Li GY, Zhang GZ, Wang YW, Li C. 2018. Complete mitochondrial genome of the bamboo snout beetle, *Cyrotrachelus buqueti* (Coleoptera: Curculionidae). Mitochondrial DNA Part B. 3(1):88–89. 33474076 10.1080/23802359.2017.1422411 PMC7800430

[CIT0003] Zhang D, Gao FL, Jakovlic I, Zou H, Zhang J, Li WX, Wang GT. 2020. PhyloSuite: an integrated and scalable desktop platform for streamlined molecular sequence data management and evolutionary phylogenetics studies. Mol Ecol Resour. 20(1) :348–355. 31599058 10.1111/1755-0998.13096

[CIT0004] Zhang SK, Shu JP, Wang YD, Liu YN, Peng H, Zhang W, Wang HJ. 2019. The complete mitochondrial genomes of two sibling species of camellia weevils (Coleoptera: Curculionidae) and patterns of Curculionini speciation. Sci Rep. 9(1):3412. 30833607 10.1038/s41598-019-39895-8 PMC6399312

